# Supplementary material for: A Systematic Review of Complementary and Alternative Veterinary Medicine: “Miscellaneous Therapies”
Source: Animals (Basel). 2021 Nov 24;11(12):3356. doi: 10.3390/ani11123356 (PMC8697896; doi:10.3390/ani11123356)
Supplement: Supplementary file 1 [file animals-11-03356-s001.zip › animals-1436236-Supplementary.pdf]

Supplementary File

A Systematic Review of Complementary and Alternative Veterinary Medicine: "Miscellaneous Therapies"

Anna Bergh, Iréne Lund, Anna Boström, Heli Hyytiäinen, and Kjell Asplund

Manual for assessment of risk of bias and relevance

Use separate templates for controlled trials (randomized or non-randomized) and observational studies.

Use the following grades: 0–5 or 1–5. For items with only three grades described (0,3,5), intermediate grades (1,2,4) may also be used.

Study design, controlled trials
1 = non-randomized controlled trial, non-adequate control group
2 = non-randomized controlled trial, adequate control group
3 = quasirandomized trial
4 = randomized controlled trial, randomization with flaws
5 = randomized controlled trial, randomization without flaws

Study design, observational studies
1 = case study
2 = cross-sectional
3 = case-control
4 = retrospective cohort
5 = prospective cohort

Statistical power
Statistical power is considered sufficient if (a) a power calculation is reported and the according number of animals have been included, *or* (b) if the principal endpoint is statistically different between groups (or corresponding):

0 = Not sufficiently powered statistically to detect an effect *or* not possible to assess.
3 = Possibly sufficiently powered to detect an effect but difficult to assess.
5 = Sufficiently powered to detect an effect.

Confounding (observational studies only)
What impact may potential confounding (e.g., concurrent other treatment) have had on the results?

0 = severe or not reported
3 = moderate
5 = none/of marginal importance

Selection/classification (observational studies only)
How well were intervention groups defined/delineated?
Was there a risk that groups were defined after the results were known?

0 = high risk of selection/classification bias or cannot be assessed

3 = intermediate risk of selection/classification bias
5 = no risk of risk of selection/classification bias

Deviation from planned therapy
This item is assessed by weighing the answers to four questions:

- Were crossovers or other deviations from planned therapy not reported?
- Was there substantial crossover (>10 percent) between intervention/control groups
- Were there other serious deviations from planned therapies?
- If deviations occurred, were they importantly unbalanced between the groups?

0 = large deviations from planned therapy *or* markedly imbalanced proportions with deviations between comparison groups *or* not reported
3 = moderate deviations from planned therapy
5 = no or only minor deviations from planned therapy

Lost to follow-up

- What proportion was lost to follow-up?
- Was loss to follow-up balanced between the groups?

0 = proportion lost to follow-up >=40% or large imbalance between groups
3 = proportion lost to follow-up 20-39%, little imbalance between groups
5 = proportion lost to follow-up 0-19%, little imbalance between groups

Outcome assessment

0 = assessor aware of what group the animals had been assigned to
3 = assessor aware of what group the animals had been assigned to but, probably, this did not have any major effect on results
5 = independent assessment of outcome (assessor unaware of treatment group, *or* laboratory, physiological or similar measurements)

If "objective" outcomes were not used, include information on who performed the assessments: the therapist (T), the animal owner (AO), blinded assessor (BA), or other (O).

Relevance
Whereas the items used for risk-of-bias scoring are about internal validity of the study, relevance is about external validity. For the sake of simplicity, it is here included as a risk-of-bias item but may also be reported separately when the systematic reviews are complied.

To what extent are the study results transferable to the Swedish setting?

0 = low relevance
3 = intermediate relevance
5 = high relevance

Overall assessment of risk of bias
A very serious bias in one item cannot be counterbalanced by high scores in other items. Therefore, the overall assessment of risk of bias is qualitative; an arithmetic summary score is only to be used as guidance.
The following risk-of-bias categories are used:

- low

- low-to-moderate
- moderate
- moderate-to-high
- high

References

Higgins J, Thomas J (eds,): Cochrane Handbook for Systematic Reviews of Interventions, version 6.2, 2021. https://training.cochrane.org/handbook/current.

Statens Beredning för Medicinsk och Social Utvärdering (SBU). Utvärdering av metoder i hälso- och sjukvården och insatser i socialtjänsten: en metodbok [in Swedish]. Stockholm 2020. Available from: http://www.sbu.se/met. Av previous version is available in English at https://www.sbu.se/globalassets/ebm/metodbok/eng_metodboken_no-longer-in-use.pdf.
